# The Australian Sheep-Goat Scale: An Evaluation of Factor Structure and Convergent Validity

**DOI:** 10.3389/fpsyg.2018.01594

**Published:** 2018-08-28

**Authors:** Kenneth Drinkwater, Andrew Denovan, Neil Dagnall, Andrew Parker

**Affiliations:** Department of Psychology, Manchester Metropolitan University, Manchester, United Kingdom

**Keywords:** belief in the paranormal, Australian Sheep-Goat Scale, Revised Paranormal Belief Scale, bifactor model, convergent validity

## Abstract

The Australian Sheep-Goat Scale (ASGS) is a commonly used measure of belief in the paranormal. The scale contains items that index extrasensory perception (ESP), psychokinesis (PK), and life after death (LAD). Although, research employs the ASGS as both a general (unidimensional) and factorial (multidimensional) measure, few studies have examined the appropriateness of these solutions. Accordingly, the present paper tested the psychometric integrity of the ASGS via two studies. Study 1 assessed ASGS factorial structure using confirmatory factor analysis. To achieve this, merging of ASGS data from previously published studies and ongoing work created a heterogeneous sample of 1,601 responses. Analysis revealed that a two-factor bifactor model best explained ASGS organization. This comprised a general overarching factor incorporating two subfactors (ESP and PK). Factor loadings and omega reliability supported a unidimensional structure for the most part. Removal of LAD items improved model fit because the factor added unnecessary complexity and undermined scale psychometric integrity. Study 2, using a supplementary composite sample of 320 respondents, assessed the convergent validity of the emergent ASGS model against a recently published Revised Paranormal Belief Scale (RPBS) bifactor solution. Comparison revealed high convergent validity. The general ASGS factor, despite deriving from only psi-related dimensions (ESP and PK) predicted RPBS scores. This finding indicated that ASGS brevity relative to the RPBS is advantageous when assessing general belief in the paranormal. The ASGS, notwithstanding limited construct content, functions as an effective measure of paranormal belief. Additionally, Study 2 replicated the bifactor structure identified in Study 1 and invariance testing supported invariance of form, factor loadings and item intercepts for this solution across Studies 1 and 2.

## Introduction

Within parapsychology, the term sheep-goat denotes believers (sheep) and disbelievers (goats) in the paranormal (Thalbourne and Haraldsson, 1980; Thalbourne and Delin, 1993). The nomenclature originated from Gertrude Schmeidler (Schmeidler, 1943, 1945; Schmeidler and McConnell, 1958), who observed that increased performance within ESP experiments was associated with the belief that ESP was possible within that particular setting. ESP in this context referred to “the non-inferential acquisition of information relating to stimuli which is inexplicable in terms of ‘orthodox’ sensory communication" (Sargent, 1981, p. 137).

Since Schmeidler’s exact use of the sheep-goat designation, the term has broadened to include belief in the possibility of ESP, experiencing ESP and acceptance of psychic phenomena generally [psychokinesis (PK) and life after death (LAD)]. More generally, researchers employ the label ubiquitously to signify the presence or absence of belief in the paranormal (Gagné and McKelvie, 1990). Thalbourne and Delin (1993) noted this generalization arose from a widening of the notion of the paranormal to include a broad spectrum of supernatural phenomena. Originally, the descriptor ‘paranormal’ served merely as a synonym for psychic.

Generic usage of the sheep-goat distinction, in part, evolved from a lack of conceptual agreement about the nature and structure of the paranormal. Hence, although measurement instruments adhere to common guidelines, such as Broad’s Basic Limiting Principles (i.e., there can be no backward causation, no action at a distance, or perception of physical events/objects unmediated by sensations) (Broad, 1949), content across scales varies significantly. For example, the Revised Paranormal Belief Scale (RPBS) (Tobacyk and Milford, 1983) characterizes the paranormal as a multidimensional construct comprising several item clusters (traditional religious belief, psi, witchcraft, superstition, spiritualism, extraordinary life forms and precognition). Contemporary psychometric assessment of the RPBS recommends a bifactor structure comprising a single overarching construct, encompassing several related, but conceptually independent subfactors (Drinkwater et al., 2017).

The presence of topics outside of core parapsychology within measurement instruments reflects the stretching and blurring of paranormal boundaries (Thalbourne and Delin, 1993). Illustratively, the Paranormal Short Inventory (Randall, 1997) contains questions about unidentified flying objects. Furthermore, scales frequently include items indexing belief in fringe subject matter, such as new age treatments (e.g., acupuncture) and lost continents (Grimmer and White, 1990). Although, these topics share thematic features with the paranormal, notably unusualness and lack of a genuine evidential basis, they are not strictly supernatural because they challenge rather than necessitate change within current scientific thinking. Acknowledging these conceptual issues, Thalbourne and Delin (1993) recommended restriction of the sheep-goat distinction to core parapsychological phenomena. This focus informed the content and development of sheep-goat measures.

Over the years, various scales have assessed the sheep-goat variable. These have differed in terms of factors and item numbers (i.e., single vs. multiple items) (Palmer, 1971, 1972). Notable examples are the Icelandic (Thalbourne and Haraldsson, 1980; Haraldsson, 1981; Haraldsson and Houtkooper, 1992) and Australian (Thalbourne and Delin, 1993) Sheep-Goat scales. The Icelandic version contains questions about belief in general extrasensory perception (ESP) [“do you believe that the existence of telepathy (thought-transference) or clairvoyance”], precognition (“do you believe that the ability to know the future or to have dreams about it is”) and frequency of reading about psychic phenomena (“do you read books or articles on psychic phenomena”). Higher scores indicate stronger belief in ESP.

The Australian Sheep-Goat Scale (ASGS) (Thalbourne and Delin, 1993) began as a list of questions assessing endorsement of ESP (belief in/experience) and LAD (the possibility of contact with the deceased) within participants taking part in an ESP experiment (Thalbourne, 1976). Subsequent item analysis produced a 10-item measure, which provided support for the sheep-goat dichotomy via correlation with experimental performance (Thalbourne and Delin, 1993). Further ESP-related work supported the scale’s predictive power (e.g., Thalbourne et al., 1982). Although, some studies observed no significant relationship between belief and performance (e.g., Thalbourne et al., 1983).

As researchers became increasingly aware of potential psychological differences between believers and non-believers the need for general measures of paranormal belief increased. For instance, researchers observed effects for locus of control (McGarry and Newberry, 1981), social interest (Tobacyk, 1983b), death threat and death concerns (Tobacyk, 1983a), dream interpretation (Haraldsson, 1981), personality (Thalbourne, and Haraldsson, 1980) and critical thinking (Alcock and Otis, 1980). Accordingly, the Paranormal Belief Scale (Tobacyk and Milford, 1983) and the 10-item ASGS (Thalbourne and Haraldsson, 1980) emerged as prevalently used measures.

In 1983, the ASGS added additional ESP items assessing belief in the possibility of precognition, sender-initiated and receptive telepathy (Thalbourne and Delin, 1993). Beyond increasing construct breadth, the 13-item version demonstrated no additional psychometric benefits; the scale correlated highly with the extant 10-item version (*r* = 0.98). The modified ASGS featured in several published studies (i.e., Irwin, 1985; Thalbourne, 1985).

In 1985, the scale appended five further items indexing belief and experience of PK. Comparisons across ASGS versions (10-, 13-, and 18-item) revealed high correlations. Psychometric evaluation of the 18-item ASGS specified the measure was a satisfactory measure of belief in and experience of core psychic phenomena. The scale demonstrated good reliability (internal and test–retest) and concurrent validity (Thalbourne and Delin, 1993). Exploratory factor analysis, using principal components analysis, confirmed that the ASGS contained three factors ESP, PK and Afterlife (Thalbourne and Delin, 1993). This built on previous analysis, which supported the notion that the ASGS was multidimensional (Thalbourne and Haraldsson, 1980; Thalbourne, 1981).

Along with variations in content, ASGS response formats have differed (i.e., forced-choice, six-point Likert and visual analog) (Thalbourne, 2010). The forced-choice format presents items as statements (e.g., “I believe in ESP”) alongside three alternative responses, “false” (zero), “uncertain” (one), and “true” (two). Summative scores range from 0 to 36, with higher scores indicating increased levels of paranormal belief (Thalbourne, 1995). The six-point Likert scale replaces fixed choices with options between “strongly disagree” and “strongly agree” (Roe, 1998). Roe (1998) advocated use of the Likert scale format because he was critical of the visual analog response style. The visual analog format, asks respondents to record level of item agreement on a horizontal line anchored by goat and sheep belief. The horizontal line is 44 units long (one unit = one-eighth if an inch). Scores on each item are obtained using a rule and range from 1 to 44; recoded as 1–10 = 0, 11–30 = 1, and 31–44 = 2. Dividing totals by 22 produced values from 0.05 to 2. Totaling across items and rounding generates scores between 1 and 36. This scoring system approximates the false, uncertain and true format (Thalbourne and Delin, 1993).

A subsequent ASGS adaptation by Lange and Thalbourne (2002) purified the scale. This involved Rasch scaling, which identified New Age-related and Traditional Belief items (LAD). Removal of the LAD items to correct for bias reduced the ASGS to 16-items. The advantage of Rasch scaling is that it produces interval-level scores, which are independent of gender and age bias. Despite these psychometric developments, the majority of studies continue to use the original 18-item measure and fail to apply Rasch scaling.

Failure to apply Rasch scaling is problematic because the two ASGS items (9 and 10) that form the LAD factor are not productive to measurement and may compromise the validity of the scale (Lange and Thalbourne, 2002). At a factorial level, Rasch scaling indicates that the LAD subscale does not function as a reliable measurement tool. Indeed, removal of the LAD factor produces a single factor New Age Belief solution (Lange and Thalbourne, 2002). This issue potentially compromises analysis at the factorial level, especially in studies, which focus on subscale differences (Rogers et al., 2016). However, at a global level, this is less of a concern because slight age and gender biases have less impact on the overall measurement of paranormal belief. Hence, original 18-item scale remains an internally reliable measure of belief in the paranormal.

The 18-item ASGS across response formats has typically demonstrated internal reliability in the good (0.80) to excellent (0.90) range (Cronbach, 1951). Illustratively, Thalbourne and Delin (1993) reported a Cronbach alpha (α) of 0.94 for the visual analog scale and Dagnall et al. (2008) an alpha of 0.92 for the forced-choice version. Foreign translations have demonstrated similar levels of internal reliability. For example, Swedish (α = 0.91) (Goulding, 2005) and Portuguese (α = 0.82) (Thalbourne et al., 2006; Thalbourne, 2010). The Rasch version of the ASGS also possesses good (α = 0.82, Dagnall et al., 2014) to excellent (α = 0.91, Storm and Rock, 2009) internal reliability. Assessing the ASGS alongside equivalent measures of belief in the paranormal (i.e., RPBS, Tobacyk and Milford, 1983; the Manchester Metropolitan University New, Dagnall et al., 2010a,b) reveals that the scale in terms of internal reliability performs at a commensurate level (Dagnall et al., 2014).

In summary, ASGS appraisal was required in order to provide conceptual clarity. Specifically, appreciation of the factorial structure of provides guidelines for the subsequent implementation and analysis of data collected via the ASGS. Accordingly, the present paper examined whether the ASGS functioned best as a multidimensional or general measure. Relative to the RPBS, few studies use ASGS subscales to assess variations in belief. However, this has occurred and informed the formation of nuanced conclusions about the functional properties of inferred dimensions (i.e., ESP, PK, and LAD) (Rogers et al., 2016). This paper tested the veracity of this approach by including multidimensionality vs. unidimensionality within a single analysis. Explicitly, bifactor modeling assessed ASGS dimensionality and factorial solution adequacy. This analysis was vital to defining the boundaries of ASGS use. In this context, previous work has failed to delineate adequately the dimensionality of the ASGS.

Noting the established use of the ASGS within the parapsychological and psychological literature and the fact that researchers report both unidimensional and factorial scores, this paper undertook two studies examining the measure’s psychometric integrity. Study 1 tested ASGS factorial structure, and Study 2, evaluated measurement invariance of the ASGS and its performance in relation to the RPBS.

## Study 1: Factorial Structure of the Australian Sheep-Goat Scale

Within published work researchers have used the ASGS as both a unidimensional (e.g., Dagnall et al., 2010a, 2011) and multidimensional measure (ESP, PK, and LAD) (Rogers et al., 2016, 2017). Although it is rare to use ASGS subscales, there are clear advantages to the inclusion of factorial comparisons. Specifically, although ESP and PK collectively represent forms of psi, the degree to which people endorse the phenomena varies. Generally, as evidenced by reported instances (Roe et al., 2003; Dagnall et al., 2016), ESP appears more plausible and probable than PK (Schmeidler, 1988; Broughton, 1991; Roe et al., 2003). In this context, it is important to assess the effectiveness of the ASGS at both a general and factorial level.

Recent related work examined the factorial structure of the RPBS (Tobacyk, 1988, 2004; Drinkwater et al., 2017). After consideration of a range of theoretically and empirically driven models, Drinkwater et al. (2017) identified the best fitting data model. This was a bifactor model comprising a single overarching construct, derived from related, but conceptually distinct subfactors. This approach usefully delimited the most appropriate scoring system for the RPBS and reaffirmed the veracity of research using general and seven-factor solutions. Accordingly, Study 1 examined whether it was valid for researchers to use both unidimensional and factorial ASGS solutions. As with the RPBS paper, the intention was to delineate appropriate scale scoring.

### Method

#### Respondents

Merging of ASGS data sets from published studies and continuing work produced a large heterogeneous sample (*N* = 1601). Several researchers have previously employed this approach to assess scale structure and integrity. Notably, evaluation of RPBS structure (Drinkwater et al., 2017), top-down purification of the RPBS (Lange et al., 2000), and Need for Closure Scale validation (Roets and Van Hiel, 2011).

Merging of ASGS data sets was appropriate because the researchers had previously used the measure in comparable studies addressing different research questions. Combining these data made it possible to examine the psychometric structure of the ASGS using sophisticated statistical techniques. Large data sets facilitate the performance of complex analytical methods by virtue of enhanced statistical power and greater within sample variation (Van der Steen et al., 2008). For these reasons, Brown (2014) advocates that confirmatory factor analysis (CFA) should use as many cases as possible. In this context, amalgamation of ASGS data sets was an expedient method that utilized existing screened data to meet these parameters. More generally, integration of small data sets avoids research costs associated with study design, recruitment and data collection, and produces a sample that would typical prove difficult to recruit because of time and cost constraints.

The mean (*M*) sample age was 27.01 years (*SD* = 11.09, range = 18–80 years). Disaggregation by gender revealed that 547 (34%) respondents were male (*M* = 28.32, *SD* = 12.81) and 1054 (66%) female (*M* = 26.48, *SD* = 11.37). Data collection occurred between September 2012 and September 2016 (see “Ethics” section). Recruitment was by emails to students (undergraduate and postgraduate) enrolled on healthcare programs (Nursing, Physiotherapy, Psychology, Speech and Language Therapy, etc.), staff across faculties at a United Kingdom university, and local businesses/community groups. There were two exclusion criteria. Firstly, respondents had to be at least 18 years of age. Secondly, in order to prevent multiple responses instructions stated that respondents must not participate if they had undertaken similar research.

#### Materials

The only measure used in Study 1 was the ASGS (Thalbourne and Delin, 1993). The ASGS assesses belief in and alleged experience of, ESP, PK, and LAD. The scale contains 18-items presented as statements. For example, “I believe in the existence of ESP” and “I believe I have marked psychokinetic ability.” Participants respond to each item on a three-point scale (false = 0, uncertain = 1, and true = 2). Raw scores range from 0 to 36, with upper scores indicating increased levels of belief in the paranormal. High scoring individuals are believers (‘sheep’) and low scorers non-believers (‘goats’). Study 1 consistent with general ASGS use included all items. The 18-item version possesses high reliability (α = 0.92) and generally performs similarly to the Rasch version (Dagnall et al., 2008).

#### Procedure

Respondents completed the ASGS in conjunction with measures assessing cognitive-perceptual personality factors, decision-making and anomalous beliefs. All studies used the same basic standardized procedures. Prior to participation, the researchers presented prospective respondents with comprehensive background information. This contained the purpose of the study and outlined ethical procedures. Respondents agreeing to participate recorded informed consent by selecting an option confirming their willingness to take part. They then received the study materials, which comprised the relevant measures and scales. Respondents also provided basic demographic information (age, preferred gender, course of study if student, etc.). Procedural instructions directed respondents to work through the sections systematically at their own pace, to answer all questions in an open and honest manner, and reassured respondents that there were no right or wrong answers. Section order rotated across respondents to prevent potential order effects.

#### Ethics

As part of the grant bidding process, the researchers obtained ethical approval for a program of studies exploring relationships between anomalous beliefs, decision-making and cognitive-perceptual personality factors (September 2012, 2014, and 2016). Each proposal was sanctioned (ethics, procedure and methodology) and rated as routine. The Director of the Research Institute for Health and Social Change (Faculty of Health, Psychology and Social Care) and Ethics Committee within *the* Manchester Metropolitan University granted ethical approval. This is the necessary level of institutional approval. Furthermore, before submission research bids are peer-reviewed by members of the Professoriate (or suitably qualified research staff). This formative process considers the appropriateness of ethics, procedures and analysis. Research proposals also receive approval from the Head of the Psychology Department.

#### Data Analysis Plan

Analysis evaluated a series of ASGS models. Firstly, a one-factor model, which acted as a baseline comparison for later solutions. Next, a correlated three-factor model based on Thalbourne and Delin (1993) examined whether ESP, PK, and LAD subfactors most effectively represented the ASGS. A bifactor version of this three-factor solution assessed the multidimensionality vs. unidimensionality assumption. Subsequently, a model based on Lange and Thalbourne (2002) tested whether a single New Age Belief factor (i.e., with Traditional Belief, LAD items 9 and 10 removed) best represented the ASGS. A correlated two-factor variant of this condensed 16-item version of the scale tested goodness of fit, with the identification of subfactors consistent with those initially proposed by Thalbourne and Delin (1993) (i.e., ESP and PK, but not LAD). Lastly, a bifactor version of this solution assessed data-model fit.

A range of indices determined goodness of fit. The chi-square (χ^2^) statistic compares the expected and observed covariance matrix, with a non-significant difference preferable. Chi-square, however, typically over-rejects good models. Accordingly, additional absolute fit indices (Root-Mean-Square Error of Approximation, RMSEA; Standardized Root-Mean-Square Residual, SRMR) assessed fit, with values of 0.05, 0.06–0.08, and 0.08–1.0 indicative of good, satisfactory, and marginal fit (Browne and Cudeck, 1993). The 90% confidence interval (CI) was included for RMSEA. Relative fit indices (Comparative Fit Index, CFI; Incremental Fit Index, IFI) compared a null with a proposed model; values above 0.90 suggest good fit (Hu and Bentler, 1999) and values above 0.86 infer marginal fit (e.g., Bong et al., 2013). Akaike’s Information Criterion (AIC) compared models with the same quantity of variables; lower values indicate superior fit.

Lastly, alpha and omega coefficients determined the reliability of the ASGS. Coefficient omega (*ω*) and omega hierarchical (*ωh*) (computed with the Omega program; Watkins, 2013) provide more effective estimates of reliability of bifactor models (Brunner et al., 2012). Coefficient omega considers specific and general factor variance in its reliability estimation, whereas hierarchical omega computes the reliability of a latent factor minus the variance from other general and specific factors.

### Results

#### Preliminary Analyses

Data screening assessing non-normality occurred prior to analysis. Skewness values were between -3 to +3, as recommended by Griffin and Steinbrecher (2013) (**Table 1**). However, Mardia’s (1970) kurtosis coefficient suggested multivariate non-normality (101.058 with a critical ratio of 75.348). Therefore, proceeding with CFA can lead to standard error biases if a correction procedure is not applied (Bentler and Wu, 2005). Analyses consequently used bootstrapping (600 resamples), which does not rely on normal data assumptions when calculating standard error estimates. Bootstrapping generates an empirical distribution related to a statistic of interest by resampling from the original data. Naïve bootstrapping performs efficiently even in situations of severe non-normality and is a robust alternative to methods including the Satorra-Bentler chi-square (Nevitt and Hancock, 2001). CFA utilized bootstrap resampling (via the bias-correction technique) to limit standard error biases and compute accurate confidence intervals at the 95% level (Byrne, 2010). The Bollen-Stine bootstrap *p* examined fit in addition to absolute and relative fit indices. Bollen-Stine is appropriate in situations where non-normality is present because it assesses fit without normal theory limitations, with *p* > 0.05 a desirable result (Bollen and Stine, 1992).

**Table 1:** Descriptive statistics and intercorrelations for ASGS total and subscales (*N* = 1601).

Variable	*Mean*	*SD*	Skew	1	2	3	4
1. ASGS total	11.90	8.34	0.55		0.96^∗∗∗^	0.75^∗∗∗^	0.62^∗∗∗^
2. Extrasensory perception	8.12	5.84	0.39			0.58^∗∗∗^	0.52^∗∗∗^
3. Psychokinesis	1.62	2.48	1.70				0.29^∗∗∗^
4. Life after death	2.16	1.42	-0.12				

^∗∗∗^*p < 0.001.*

An inspection of intercorrelations revealed that the ASGS total score and the subfactors of ESP, PK, and LAD possessed significant positive relationships. A large correlation of 0.96 existed between ASGS total and ESP, which is reflective of the fact that ESP comprises a large number of ASGS items (10). The lowest intercorrelation existed between PK and LAD, *r* (1599) = 0.29, *p* < 0.001.

#### Confirmatory Factor Analysis

The one-factor model reported a poor fit, χ^2^ (135, *N* = 1601) = 4539.67, *p* < 0.001, CFI = 0.66, IFI = 0.66, RMSEA = 0.14 (90% CI of 0.14 to 0.15), SRMR = 0.10. Bollen-Stine, *p* = 0.002, also indicated poor fit. The correlated three-factor model (Thalbourne and Delin, 1993) suggested unsatisfactory fit on all indices, χ^2^ (131, *N* = 1601) = 2310.88, *p* < 0.001, CFI = 0.83, IFI = 0.83, RMSEA = 0.10 (90% CI of 0.09 to 0.11), but SRMR = 0.80. Bollen-Stine, *p* = 0.002, indicated poor fit. A three-factor bifactor model reported marginal fit, χ^2^ (117, *N* = 1601) = 1746.75, *p* < 0.001, CFI = 0.88, IFI = 0.88, RMSEA = 0.09 (90% CI of 0.08 to 0.10), SRMR = 0.06. Bollen-Stine, *p* = 0.002, indicated poor fit. A lower AIC for the three-factor bifactor solution (AIC = 1890.75) existed in comparison with one-factor (AIC = 4647.67) and correlated three-factor (AIC = 2426.88) variants, suggesting the bifactor was most suitable for the data.

**FIGURE 1:**
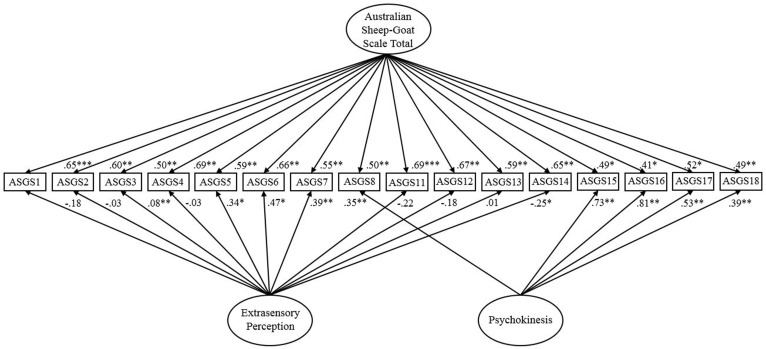
Two-factor bifactor model of the Australian Sheep-Goat Scale. Latent variables are represented by ellipses; measured variables are represented by rectangles; error is not shown but was specified for all variables. ^∗^*p* < 0.05; ^∗∗^*p* < 0.01; ^∗∗∗^*p* < 0.001 (using bootstrap significance estimates).

The one-factor model based on Lange and Thalbourne (2002) fitted poorly overall, χ^2^ (104, *N* = 1601) = 3861.74, *p* < 0.001, CFI = 0.68, IFI = 0.68, RMSEA = 0.15 (90% CI of 0.14 to 0.16), SRMR = 0.10. Bollen-Stine, *p* = 0.002, supported poor fit. A correlated two-factor version of Lange and Thalbourne (2002) reported unsatisfactory fit on all indices, χ^2^ (102, *N* = 1601) = 2102.99, *p* < 0.001, CFI = 0.83, IFI = 0.83, RMSEA = 0.11 (90% CI of 0.10 to 0.12) but SRMR = 0.08. Bollen-Stine, *p* = 0.002, supported unsatisfactory fit. A two-factor bifactor solution (**Figure 1**) indicated good fit on all indices, χ^2^ (86, *N* = 1601) = 1196.78, *p* < 0.001, CFI = 0.91, IFI = 0.91, SRMR = 0.06, but RMSEA = 0.09 (90% CI of 0.08 to 0.09) suggested marginal fit. Bollen-Stine, *p* = 0.002, supported poor fit. However, given marginal to good fit existed on absolute and relative indices, it is possible the Bollen-Stine result is symptomatic of the large sample (Cooper, 2017). Accordingly, analysis considered the Standardized Residual Covariance Matrix. The majority of residual covariances should be less than two if the model signifies good fit (Joreskog, 1993), this was apparent for approximately 85% of cases. Consultation of AIC across the 16-item solutions suggested that the bifactor variant (AIC = 1330.78) fitted these data more suitably than the one-factor (AIC = 3957.74) and correlated two-factor model (AIC = 2202.99). Compared with the three-factor bifactor model, the two-factor bifactor solution possessed marginally superior data-model fit. This therefore was the most appropriate factorial conceptualization of the ASGS.

An inspection of factor loadings for the two-factor bifactor model revealed that all items loaded above the minimum cut-off of 0.32 (recommended by Tabachnick and Fidell, 2001) on the general ASGS factor. In comparison, items 3, 4, 2, and 13 did not significantly load on the ESP subfactor, indicating that these items more directly predicted a general factor. In addition, negative loadings were evident for some ESP items, which can unexpectedly occur in bifactor models (e.g., Toplak et al., 2009; Chen et al., 2012) as a function of a crossover suppression effect (Paulhus et al., 2004). The average factor loadings were 0.56 on PK, 0.03 on ESP, and 0.58 on the general ASGS factor. Although loadings were satisfactory for PK, items 15, 16, 17 loaded more highly than on the general factor. Interestingly, these items index personal experience of PK, which are slightly different in tone to the rest of the measure that focuses more on belief. Overall, belief within the ASGS best represented a general factor, particularly in relation to ESP items. However, items related to PK also require consideration. The poor data-model fit of the one-factor 16-item solution reinforces this finding.

#### Reliability

The 16-item ASGS possessed high reliability (α = 0.89, 95% CI of 0.86 to 0.88). Reliability was high for both the PK (α = 0.85, 95% CI of 0.84 to 0.86) and ESP (α = 0.87, 95% CI of 0.86 to 0.90) subfactors. For completeness, assessment of the 18-item ASGS reported high reliability (α = 0.90, 95% CI of 0.89 to 0.91). Internal consistency was slightly lower for LAD (α = 0.69, 95% CI of 0.66 to 0.72); however, this was close enough to the threshold of 0.70 to be deemed acceptable. In addition, Nunnally and Bernstein (1978) suggest that an alpha above 0.60 is satisfactory within psychological science.

Consistent with alpha, coefficient omega reported high reliability for a general ASGS factor (*ω* = 0.92), PK (*ω* = 0.87), and ESP (*ω* = 0.89). Omega hierarchical was reasonably high for a general ASGS factor (*ωh* = 0.84). Lower estimates existed for PK (*ωh* = 0.51) and ESP (*ωh* = 0.01). A general ASGS factor explained 69% of the variance. PK and ESP accounted for 22.3% and 8.7%, respectively. The percentage of uncontaminated correlations (PUCs) was 45.8%, signifying a reasonable number of correlations reflect general factor variance.

### Discussion

Study 1 found that a two-factor bifactor model best represented ASGS measurement. This comprised a general overarching factor encompassing two related, but conceptually independent subfactors (ESP and PK). Omega reliability supported this conceptualization. However, an important degree of variance existed for ASGS subfactors, particularly PK. The poor data-model fit of the one-factor solution reinforced the validity of the two-factor bifactor solution. Therefore, it is likely that the ASGS reflects a unidimensional structure for the most part, but specific PK items need consideration when implementing the measure. Concurring with Lange and Thalbourne (2002), analysis suggested that the New Age-related items best measured belief in the paranormal. Removal of LAD items, indexing Traditional Belief, improved model fit. These items added unnecessary complexity to the model.

Omission of the LAD subscale is conceptually and psychometrically appropriate because the subscale comprises only two items. Accordingly, LAD fails to assess adequately construct breadth. Additionally, informed advocates of paranormal powers class ESP and PK as forms of psi. This notion derives from the supposition that ESP and PK arise from inexplicable forces beyond the understanding of established physical principles. Hence, there is considerable parapsychological debate about the extent to which the two alleged phenomena share common features and/or overlap (Roe et al., 2003). Some theorists have gone so far as to question whether ESP and PK represent a unitary domain (Schmeidler, 1988, 1994). Whereas, other theorists view the two phenomena as complementary, but distinct in terms of characteristics and predisposing conditions. This notion accords with layperson accounts of psi. Thus, although the phenomena share considerable variance, individuals more commonly report ESP experiences and people generally consider the existence of ESP more likely than PK (Storm and Thalbourne, 2005).

Contrastingly, LAD represents a distinct belief dimension. The RPBS, despite issues with factorial structure, acknowledges this distinction. Explicitly, LAD within the RPBS straddles two separate factors, traditional religious belief (i.e., “The soul continues to exist though the body may die”) and spiritualism (i.e., “It is possible to communicate with the dead”).

These are distinct to the psi subscale, which comprises a combination of PK (3) and ESP (1) items, respectively.

Overall, Study 1 indicated that the ASGS functions well at a global level. The original two-factor solution (with LAD omitted) was the best fitting bifactor model.

## Study 2: Invariance and Convergent Validity of the Australian Sheep-Goat Scale

The RPBS due to its breadth and multidimensionality features in a range of empirical research. Indeed, the RPBS is the most widely used measure of paranormal belief within psychology (Goulding and Parker, 2001). Conversely, parapsychologists tend to use the ASGS because it focuses on fundamental paranormal concepts (extra-sensory perception, PK and LAD). The preference for the ASGS stems from the fact that the measure evolved within parapsychology, where it subsequently developed an acknowledged reputation.

The fact that psychology and parapsychology often use different measures to assess belief in the paranormal creates potential issues. Primarily, the concern that results may arise as an artifact of the scale used and may not extrapolate across studies. For these reasons, it is important to ensure that the RPBS and ASGS index the same underlying construct. This is not easy to establish because few studies have used the scales concurrently. When employed together, studies report high positive correlations between the two measures (Drinkwater et al., 2012; Dagnall et al., 2014). Indeed, the scales share approximately 60% variance. This suggests that the RPBS assesses the core aspects of the paranormal belief indexed by the ASGS (ESP, LAD, and PK). Convergent validity testing will examine the degree of convergence between the ASGS and RPBS.

An additional method that tests whether the ASGS is transferable across studies is invariance testing. This assesses whether measurement interpretation is consistently across contexts and populations (and hence differences reflect true mean variations) or if any observed differences in scores are likely due to an artifact of the measure (Wu et al., 2007). A paucity of research has examined invariance of the ASGS.

Study 2 examined measurement invariance and the convergent validity of the ASGS bifactor model specified in Study 1. Specifically, following analysis of factor structure invariance tests examined whether the bifactor solution was invariant across each of the samples used (i.e., for Studies 1 and 2). Evaluating invariance among samples from separate studies is consistent with the approach of Schellenberg et al. (2014), and determines whether the ASGS is trustworthy across different studies.

Furthermore, using structural equation modeling (SEM) in a latent modeling context, Study 2 evaluated the extent to which ASGS scores predicted the RPBS bifactor solution identified by Drinkwater et al. (2017). This was important because convergence specifies the extent to which the scales measure general paranormal belief and share conceptual overlap (ESP and PK). In addition, analysis via SEM is preferable to alternative approaches, such as regression analysis, because SEM incorporates a simultaneous assessment of latent constructs and measurement error when assessing relationships (Blanthorne et al., 2006). This provides a more parsimonious evaluation of the shared variance among variables. Another advantage of SEM is the assessment of the measurement models in a confirmatory manner prior to testing structural relationships. By focusing on the degree of consistency between data and proposed factor model, SEM offers a rigorous assessment of the scales. This approach ensures that prior to testing structural relationships measures are reliable and fit with the existing *a priori* conceptions.

### Method

#### Respondents

The sample comprised 320 respondents who had completed both the ASGS and the RPBS. Respondents mean (*M*) age = 29.57 (*SD* = 11.09, range = 18–62 years). Within the sample 95 (30%) were male (*M* = 30.94, *SD* = 13.35) and 225 (70%) female (*M* = 29.00, *SD* = 11.96). Respondents were collected following the perimeters outlined in Study 1. Since the original studies looked at a range of beliefs over a period of time instructions told respondents not to participate if they had previously completed research on belief in the paranormal. This direction ensured that Studies 1 and 2 contained different respondents.

#### Measures

Study 2 used the 16-item version of the ASGS tested in Study 1 and the RPBS (Tobacyk and Milford, 1983). The RPBS contains 26-items that assess belief in the paranormal. Respondents indicate level of agreement to statements (e.g., “There is a devil”) on a seven-point Likert scale (responses range from 1 = strongly disagree to 7 = strongly agree). The RPBS comprises seven belief dimensions: Traditional Religious Belief (TRB), Psi Belief (PSI), Precognition (PRE), Superstition (SUP), Witchcraft (WIT), Spiritualism (SPIR), and Extraordinary Lifeforms (ELF). Previous research documents that the RPBS has adequate reliability and validity. Specifically, Drinkwater et al. (2017) reported a Cronbach alpha of 0.93 (95% CI of 0.92 to 0.93) for the full scale. The subscales were also satisfactorily reliable (apart from ELF). Specifically, TRB α = 0.88 (95% CI of 0.87 to 0.89); WIT α = 0.80 (95% CI of 0.79 to 0.81); PSI α = 0.83 (95% CI of 0.82 to 0.83); SUP α = 0.83 (95% CI of 0.82 to 0.84); SPIR α = 0.83 (95% CI of 0.82 to 0.84); PRE α = 0.86 (95% CI of 0.85 to 0.87). ELF indexed lower reliability, as α = 0.54 (95% CI of 0.52 to 0.57). Additional existing studies report concerns with the ELF subscale (e.g., see Lawrence, 1995), and this is a commonly accepted issue with the RPBS. Accordingly, theorists generally regard the RPBS as a satisfactory measure of belief in the paranormal overall (Tobacyk, 2004). Some critics question the dimensionality of the measure. A recent paper by Drinkwater et al. (2017) recommends a bifactor solution, where the RPBS best represents belief in the paranormal as a general overarching construct comprising several related, but conceptually independent subfactors.

#### Procedure and Ethics

Procedure and ethics were identical in Study 1. As in previous projects, when respondents completed the ASGS and RPBS together, scale presentation order was counterbalanced.

#### Data Analysis Plan

Prior to assessing structural relationships, CFA examined the two-factor bifactor ASGS model from Study 1 and the bifactor RPBS model independently. Alpha and omega coefficients determined scale reliability. Multi-group CFA assessed invariance in relation to progressively restrictive models among the sample from Study 1 (*N* = 1601) and Study 2 (*N* = 320). Models tested invariance at the configural (invariance of form or factor structure), metric (invariance of factor loadings) and scalar (invariance of item intercepts) level. This comprises an assessment of configural, weak and strong factorial invariance (Meredith, 1993). Further tests (i.e., strict invariance) are unnecessary given these are rarely satisfied (Byrne, 2010). Critical values using Chen’s (2007) criteria determined suitable fit at each stage: a CFI difference below 0.01 alongside an RMSEA difference less than 0.015. Invariance did not consider chi-square due to its sensitivity with large samples, as recommended by Brown (2014).

To examine the convergent validity of the ASGS, both specific and general factors of the bifactor model were regressed onto the RPBS. Specifically, the established seven-factor bifactor solution of Drinkwater et al. (2017) acted as the criterion. In this model, a general factor of paranormal belief explained the majority of RPBS variance; therefore, the general factor acted as the dependent variable while controlling for the variance of the seven factors. The relative strength of general vs. specific subfactors of the ASGS in relation to the RPBS indicates which facets most appropriately align with an established index of paranormal belief. Analysis considered identical model fit indices to Study 1.

### Results

#### Preliminary Analyses

Skewness scores were within the range of -3 to +3 (**Table 2**). However, examination of Mardia’s (1970) kurtosis coefficient revealed multivariate non-normality: ASGS (132.34, critical ratio = 49.32), RPBS (184.44, critical ratio = 43.24), and both scales in conjunction (423.56, critical ratio = 62.32). As with Study 1, analyses used bootstrapping (600 resamples) to generate confidence intervals at the 95% level (bias-corrected) (Byrne, 2010). The Bollen-Stine bootstrap *p* additionally examined fit.

**Table 2:** Descriptive statistics and intercorrelations for ASGS total, RPBS total, and subscales (*N* = 320).

Variable	*Mean*	*SD*	Skew	1	2	3	4	5	6	7	8	9	10	11
1. ASGS total	10.04	7.61	0.61		0.97^∗∗∗^	0.71^∗∗∗^	0.77^∗∗∗^	0.49^∗∗∗^	0.67^∗∗∗^	0.58^∗∗∗^	0.37^∗∗∗^	0.40^∗∗∗^	0.74^∗∗∗^	0.78^∗∗∗^
2. Extrasensory perception	7.08	5.50	0.47			0.56^∗∗∗^	0.71^∗∗∗^	0.39^∗∗∗^	0.63^∗∗∗^	0.56^∗∗∗^	0.33^∗∗∗^	0.40^∗∗∗^	0.69^∗∗∗^	0.71^∗∗∗^
3. Psychokinesis	0.96	1.73	2.07				0.47^∗∗∗^	0.34^∗∗∗^	0.44^∗∗∗^	0.29^∗∗∗^	0.24^∗∗∗^	0.19^∗∗^	0.44^∗∗∗^	0.50^∗∗∗^
4. RPBS total	49.60	30.41	0.36					0.73^∗∗∗^	0.79^∗∗∗^	0.79^∗∗∗^	0.62^∗∗∗^	0.56^∗∗∗^	0.88^∗∗∗^	0.88^∗∗∗^
5. Traditional religious belief	10.29	7.28	0.20						0.40^∗∗∗^	0.53^∗∗∗^	0.35^∗∗∗^	0.26^∗∗∗^	0.52^∗∗∗^	0.58^∗∗∗^
6. Psi beliefs	7.24	5.55	0.76							0.58^∗∗∗^	0.40^∗∗∗^	0.37^∗∗∗^	0.68^∗∗∗^	0.71^∗∗∗^
7. Witchcraft	7.51	6.76	0.66								0.34^∗∗∗^	0.42^∗∗∗^	0.61^∗∗∗^	0.60^∗∗∗^
8. Superstition	3.43	4.25	0.39									0.35^∗∗∗^	0.58^∗∗∗^	0.43^∗∗∗^
9. Extraordinary lifeforms	5.95	3.36	0.42										0.45^∗∗∗^	0.44^∗∗∗^
10. Precognition	7.30	5.78	0.35											0.79^∗∗∗^
11. Spirituality	7.84	6.57	0.50											

^∗∗∗^*p < 0.001.*

Significant intercorrelations existed among all variables, with the lowest correlation between Extraordinary Lifeforms and PK, *r* (318) = 0.19, *p* = 0.001. Similar to Study 1, a high correlation existed between ESP and ASGS, *r* (318) = 0.98, *p* < 0.001. The highest correlation among RPBS and ASGS subfactors existed in relation to ESP and Spirituality, *r* (318) = 0.71, *p* < 0.001. ASGS and RPBS correlated positively, *r* (318) = 0.71, *p* < 0.001.

#### Confirmatory Factor Analysis and Reliability

Analysis via CFA reported good fit across all indices for the two-factor bifactor ASGS solution, χ^2^ (86, *N* = 320) = 279.78, *p* < 0.001, CFI = 0.91, IFI = 0.91, RMSEA = 0.08 (90% CI of 0.07 to 0.09), SRMR = 0.06. Bollen-Stine, *p* = 0.002, suggested poor fit. However, the majority of standardized residual covariances were below 2. The seven-factor bifactor RPBS solution reported good data-model fit, χ^2^ (270, *N* = 320) = 677.120, *p* < 0.001, CFI = 0.93, IFI = 0.93, RMSEA = 0.07 (90% CI of 0.06 to 0.08), SRMR = 0.06. To maintain consistency with Drinkwater et al. (2017) analysis permitted error covariance between items 1 and 15, 3, and 17, 21 and 26. Bollen-Stine, *p* = 0.002, inferred poor fit. However, the majority of standardized residual covariances were below 2, suggesting that the estimated model fitted these data well.

Similar to Study 1, ASGS items loaded reasonably well on a general factor (i.e., *p* < 0.05) with an average loading of 0.54. In addition, items loaded sufficiently on PK (average loading of 0.51) and lower on ESP (average loading of -0.08). Consistent with Study 1, items 15, 16, 17 loaded highly on PK and to a greater degree than the general factor. Negative loadings were again apparent for ESP. This subfactor was more complex to interpret in the presence of a general ASGS factor. It is important to note that these negative loadings occurred in the context of positive, significant loadings on a general ASGS factor. Relatedly, all RPBS items loaded relatively highly on a general factor (all above 0.32 with *p* < 0.05) apart from item 20 (loading = 0.31). These results infer satisfactory replication of the bifactor factorial structure of the ASGS and the RPBS.

Alpha reliability of ASGS (α = 0.88, 95% CI of 0.86 to 0.90) and ESP (α = 0.87, 95% CI of 0.85 to 0.89) was high. Reliability of PK (α = 0.76, 95% CI of 0.71 to 0.80) was satisfactory. Coefficient omega was also high for ASGS (*ω* = 0.91), PK (*ω* = 0.81), and ESP (*ω* = 0.88). Omega hierarchical was high for a general ASGS factor (*ωh* = 0.83), with lower results for PK (*ωh* = 0.50) and ESP (*ωh* = 0.02). A general factor accounted for 68.1% of common variance, with PK and ESP explaining 2.25% and 0.93%, respectively. Consistent with Study 1, a reasonable number of correlations reflected general factor variance (PUC = 45.8%).

Internal consistency of RPBS total was high (α = 0.94, 95% CI of 0.93 to 0.95). Similarly, reliability was high for RPBS subscales: TRB (α = 0.88, 95% CI of 0.85 to 0.90), SUP (α = 0.84, 95% CI of 0.81 to 0.87), WIT (α = 0.88, 95% CI of 0.86 to 0.90), SPIR (α = 0.87, 95% CI of 0.85 to 0.89), and PRE (α = 0.84, 95% CI of 0.81 to 0.87). Reliability was satisfactory for PSI (α = 0.79, 95% CI of 0.75 to 0.83). ELF possessed an alpha below 0.60 of 0.59 (95% CI of 0.50 to 0.66). This result was consistent with previous research concerning psychometric properties of the RPBS (Drinkwater et al., 2017). Omega reported similarly high reliability for RPBS (*ω* = 0.97), TRB (*ω* = 0.89), PSI (*ω* = 0.84), WIT (*ω* = 0.89), SUP (*ω* = 0.85), SPIR (*ω* = 0.89), and PRE (*ω* = 0.88). ELF was lower (*ω* = 0.68). A high omega hierarchical coefficient existed for a general RPBS factor (*ωh* = 0.89) compared with TRB (*ωh* = 0.50), PSI (*ωh* = 0.24), WIT (*ωh* = 0.38), SUP (*ωh* = 0.57), SPIR (*ωh* = 0.06), PRE (*ωh* = 0.11), and ELF (*ωh* = 0.44). The general factor explained 59.2% of common variance and the PUCs was high (PUC = 88.9%) supporting the superiority of a general factor.

#### Multi-Group Analysis

Invariance testing used bootstrapping (600 resamples) and the Bollen-Stine *p*-value due to the presence of data non-normality across Studies 1 and 2. Mardia’s (1970) coefficient supported this decision (99.14, critical ratio = 82.64). Assessment of configural invariance for the bifactor ASGS model across Study 1 and Study 2 suggested satisfactory data-model fit, χ^2^ (172, *N* = 1921) = 1724.92, *p* < 0.001, CFI = 0.89, IFI = 0.89, RMSEA = 0.06 (90% CI of 0.06 to 0.07), SRMR = 0.06. A metric invariance test (CFI = 0.88, RMSEA = 0.06) reported an acceptable CFI difference of 0.006 and an RMSEA difference of 0.003. Assessment of scalar invariance (CFI = 0.88, RMSEA = 0.06) supported strong factorial invariance (CFI difference = 0.002, RMSEA difference = 0.002). Bollen-Stine *p* = 0.002, yet 85% of standardized residual covariances were lower than 2.

#### Model Test

**FIGURE 2:**
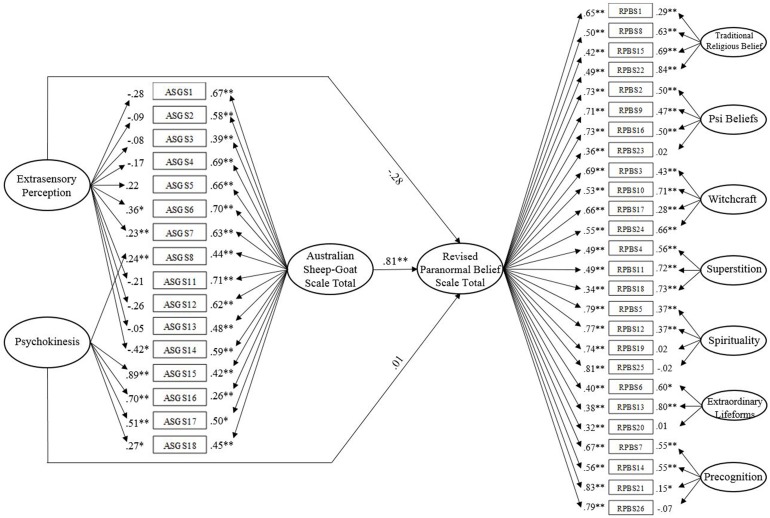
Convergent validity of the Australian Sheep-Goat Scale. Latent variables are represented by ellipses; measured variables are represented by rectangles; error is not shown but was specified for all variables. Error covariances between RPBS1 and RPBS15, RPBS3 and RPBS17, RPBS21 and RPBS26 are not shown but were included. ^∗^*p* < 0.05; ^∗∗^*p* < 0.01 (using bootstrap significance estimates).

A full structural test of the linear relationship between the bifactor ASGS model and the bifactor RPBS model (**Figure 2**) reported satisfactory data-model fit, χ^2^ (771, *N* = 320) = 1799.54, *p* < 0.001, CFI = 0.89, IFI = 0.89, RMSEA = 0.06 (90% CI of 0.06 to 0.07), SRMR = 0.06. Bollen-Stine, *p* = 0.002, suggested poor fit. However, the majority of standardized residual covariances were below 2. An examination of structural paths revealed that the ASGS general factor (ASGS) significantly predicted the general RPBS factor while accounting for the variance of the seven RPBS subfactors, β = 0.81, *p* = 0.002 (95% CI of 0.66 to 0.89). ESP and PK, however, did not significantly predict RPBS, β = -0.28, *p* = 0.19 (95% CI of -0.57 to 0.14) and β = 0.01, *p* = 0.84 (95% CI of -0.09 to 0.12), respectively. The model accounted for 73.9% of variance in RPBS.

The next model in order to examine the effect of ASGS subfactors on RPBS, constrained the path from ASGS to RPBS to zero. The constrained solution reported marginal fit on all indices χ^2^ (772, *N* = 320) = 1799.54, *p* < 0.001, CFI = 0.88, IFI = 0.88, RMSEA = 0.06 (90% CI of 0.06 to 0.07) but SRMR = 0.10, indicated unacceptable fit. Inspection of the structural paths indicated that ESP and PK significantly predicted RPBS in the absence of ASGS, β = 0.87, *p* < 0.001 (95% CI of 0.80 to 1.04) and β = 0.23, *p* = 0.002 (95% CI of 0.08 to 0.36), respectively. AIC values (in addition to the fit indices mentioned above) specified that the full model (AIC = 2056.46) possessed a superior data-model fit compared with the constrained model (AIC = 2143.54). In addition, Bollen-Stine *p* = 0.002. Compared with the full model, a considerably greater number of residual covariances were higher than 2, inferring that the full model estimated these data more appropriately. Results supported the superiority of a general ASGS factor in predicting a related criterion (RPBS). Due to successful replication with a separate sample, findings established convergent validity of the bifactor ASGS structure. Particularly, they provide evidence of a strong relationship with a comparable measure of paranormal belief.

### Discussion

Study 2, revealed high convergent validity between the ASGS and RPBS bifactor solutions. The general ASGS factor despite deriving only from psi related dimensions (ESP and PK) predicted RPBS scores. This suggested that the brevity of the ASGS in relation to the RPBS is a distinct advantage when assessing general paranormal belief. The ASGS despite limited construct content appears to function as an effective measure of paranormal belief. A discrepancy relates to the negative relationship between ESP and RPBS in the full model. As documented in previous research (e.g., Chen et al., 2012), the patterns of predictive relations in a bifactor analysis can be the opposite of zero-order correlations. ESP and RPBS possessed an initial *r* = 0.71. This discrepancy is likely a function of the general ASGS factor assuming variance in the analysis given the relationship between ESP and RPBS emerged as positive after controlling for ASGS.

Additionally, Study 2 replicated the factorial structure identified in Study 1 with a discrete sample of respondents. Particularly, a general ASGS factor accounted for the majority of scale variance and possessed a similar magnitude of average factor loading. PK demonstrated reasonably high factor loadings, and (as with Study 1) ESP recorded a low average factor loading, suggesting that the majority of items likely predict general ASGS. Omega hierarchical estimates supported these results. Measurement invariance tests comparing Study 1 with Study 2 respondents supported configural, weak and strong invariance for the bifactor ASGS solution. Findings confirmed that the bifactor ASGS model was robust and, to an extent, generalizable. Study 2 also successfully replicated the seven-factor bifactor RPBS solution of Drinkwater et al. (2017).

## Overall Discussion

Assessment of ASGS structure revealed that a two-factor bifactor model, comprising a general paranormal belief dimension encompassing two discrete but related facets (ESP and PK), demonstrated superior fit. Factor loadings were higher for a general factor and hierarchical omega indicated that a general factor accounted for the majority of variance. These results support the notion that the ASGS measures general level of paranormal belief and is, for the most part, unidimensional (Lange and Thalbourne, 2002). In practice, findings recommend the use of total scale scores as opposed to independent subscales. An amount of non-redundant variance existed, however, particularly for PK. In addition, ESP and PK subscales were conceptually compatible with their factor labels and demonstrated reliability. These subscales can be utilized when administering the measure, but in the presence of general scale scores. This conclusion is consistent with other published work relating to bifactor models (e.g., McElroy et al., 2018). Invariance testing supported invariance of form, factor loadings and item intercepts across both Studies 1 and 2. These results are encouraging because they suggest that differences in ASGS scores are likely to reflect true mean variations rather than measurement bias, thus supporting future use of the measure across different subpopulations/samples.

From a belief measurement perspective, the emergent model was congruent with the view that ESP and PK denote associated forms of phenomena. Indeed, based on global taxonomic features, parapsychology classifies both as forms of psi. Conceptual overlap arises from the fact that ESP and PK arise from alleged psychic/mental powers, whose existence contravenes established scientific principles. These characteristics apply also to other phenomena. Hence, the term psi embraces further paranormal occurrences, such as precognition and remote viewing (Irwin and Watt, 2007). Consideration of individual psi facets reveals that they vary greatly in terms of credibility. This is important in the context of the ASGS because previous empirical work reveals that people generally believe that ESP is more plausible and probable than PK (Schmeidler, 1988; Broughton, 1991; Roe et al., 2003). Higher endorsement rates and reported instances evidence this (Roe et al., 2003; Dagnall et al., 2016).

Contrastingly, LAD items do not relate to psychic/mental powers. Instead, they assess belief in spirits and the afterlife. These notions draw on elements of religious belief and spiritualism as acknowledged by the RPBS (Tobacyk, 1988). This distinction is also apparent within Lange and Thalbourne’s (2002) Rasch scaling of the ASGS, which identified a New Age-related factor comprising PK and ESP, and a Traditional Belief dimension composed of LAD items. Consistent with Lange and Thalbourne (2002), the present paper found that exclusion of the LAD or Traditional Belief dimension (ASGS items 9 and 10) improved model fit. The LAD subscale undermined scale integrity by adding unnecessary complexity to the measure.

Overall, the present findings supported the veracity of previous research, which has used the ASGS as an overall and factorial measure of belief in the paranormal. However, conclusions derived from the LAD (Rogers et al., 2016, 2017) require further evaluation and replication with more reliable measures. Alternatively, in order to increase LAD construct breadth and subscale integrity researchers could generate and assess the effectiveness of additional afterlife-related items. The LAD subscale focuses particularly on the survival hypothesis. This is a broad construct, which necessitates consideration of related concepts, such as religious beliefs and spiritualism. The RPBS acknowledges this. Additionally, reference to ghosts and hauntings would prove useful because these subjects are associated with the afterlife and represent commonly endorsed paranormal beliefs and frequently reported experiences (Gallup and Newport, 1990; Newport and Strausberg, 2001). Given the importance of these phenomena it is odd that they are absent from both the ASGS and RPBS (Dagnall et al., 2010b).

The current study suggests that the ASGS and the RPBS are of equal importance. The notion that the ASGS is superior to the RPBS is unjustified. Rogers et al. (2009) claimed that content and psychometric issues undermined RPBS validity. The present paper found that these measures demonstrated good convergent validity. Both index general paranormal belief and function as equivalent measures. This finding corresponded with Dagnall et al. (2014), who demonstrated that the ASGS and RPBS produced comparable findings when assessing relationships between belief in the paranormal and susceptibility to probabilistic biases. Clearly, the present results support the notion that outcomes do not vary as a function of using the ASGS or RPBS. This seems obvious, but given the previously published criticism, is an important result worth noting. Accordingly, researchers should feel confident when using either measure of paranormal belief.

### Limitations

Within the present paper, both studies assessed belief in the paranormal at only one point in time. Study 1 evaluated ASGS structure via completion of the measure, and Study 2, used a cross-sectional design to further assess ASGS structure and examine convergent validity between the ASGS and RPBS. This approach was potentially problematic because scores may vary over time. In the context of the current study, this was less of a concern because paranormal beliefs generally remain temporally stable (Kim et al., 2015). Additionally, both the ASGS (Thalbourne and Delin, 1993) and RPBS (Tobacyk, 2004) have previously demonstrated satisfactory test–retest reliability indicating that scores remain relatively unchanging. Concerning ASGS structure, Study 2 replicated the model found in Study 1, indicating that the two-factor bifactor was robust and replicable within an independent sample. Future studies may wish to assess further temporal stability by testing respondents on two different occasions. In addition, the same items loaded highly on PK (15, 16, 17) relative to a general factor across the two studies. Therefore, it would be beneficial for future research to consider controlling for these items (e.g., as a method factor) when utilizing the ASGS.

From a psychometric perspective, the ASGS possesses limitations similar to the RPBS. Particularly, potential response bias arising from an overreliance on positively phrased statements. Typically, scale designers add negative particles (reverse orienting), or use words with an opposite meaning (reverse wording) to counter the tendency to agree to questions (acquiescence bias) or select extreme options (extreme responses) (Van Sonderen et al., 2013). Although, test developers view this as good practice (Baumgartner and Steenkamp, 2001), in the context of belief scales reversed items are highly problematic (Drinkwater et al., 2017). Specifically, they are difficult to comprehend and failure to endorse specific items does not indicate disbelief. With complex notions such as ESP, rejecting particular instances does not necessarily indicate lack of belief in the general domain. For example, a person may not believe it possible to predict the future via dreams, but may consider that visions and feelings can foretell forthcoming events. For these reasons, positively worded items that represent the degree to which respondents endorse the existence of phenomena appear adequate. Indeed, the performance of the one reversed RPBS item (question 23) provides support for this contention. This question typically performs poorly in comparison to other scale items (with the exception of the extraordinary life forms subscale).

The problem of reversing items generally is that respondents often miss negative content. This is especially true when a reversed statement is located within a block of standardly phrased items (Drolet and Morrison, 2001). This occurs because inattention can produce misappropriate item grouping. This may explain why reversed items frequently load on separate factors and fail to confirm to general factorial models (Herche and Engelland, 1996). These issues are consistent with the conclusion of Van Sonderen et al. (2013), who contend that reversing items is a counterproductive strategy that results in response contamination arising from respondent inattention and confusion.

The ASGS is one of the most commonly used measures of paranormal belief, it is therefore essential that researchers appreciate the measure’s advantages and limitations. The present study is important because it provides the background for and an overview of the ASGS. Concomitantly, this article advises on scoring and interpretation. Recently, similar work with the RPBS produced conceptual clarity and an implementation framework (Drinkwater et al., 2017). In this context, analysis suggests that the measure functions best as an overall measure of paranormal belief.

Generally, the study of paranormal belief is of value because such beliefs are widely held within society (Irwin, 1993), they are an important aspect of the human condition (Irwin, 2009), and believers (vs. non-believers) demonstrate important differences (psychological, pathological, sociological, etc.) (Irwin, 1993). These may influence the way people think, perceive, interpret, and comprehend the world. Hence, the investigation of paranormal belief affords insights into everyday cognitions and behaviors and in doing so contributes to several academic disciplines (e.g., psychology, parapsychology, neuroscience, physics, engineering, and biology).

At a practical level, research on paranormal belief informs understanding of psychological functioning and well-being (Irwin, 2009). Accordingly, well-designed measures help researchers to identify correlates of beliefs, and in doing so advise the development of complex multivariate models. Recently, these have usefully combined belief with cognitive-perceptual personality factors and preferential thinking style. This approach has resulted in the emergence of dual influence models that apply to other beliefs and behaviors (Denovan et al., 2017). Additionally, this approach has been highly productive in promoting understanding of scientifically unsubstantiated beliefs generally (i.e., conspiracy theory, Dagnall et al., 2015; and urban legends, Dagnall et al., 2017).

## Author Contributions

KD: theoretical focus and analysis, design, background, and data collection. AD: theoretical focus and analysis, analysis and model testing. ND: theoretical focus and analysis, contributed to and supported all sessions. AP: commented on drafts—provided theoretical background and draft feedback.

## Conflict of Interest Statement

The authors declare that the research was conducted in the absence of any commercial or financial relationships that could be construed as a potential conflict of interest.
